# *Akkermansia muciniphila* Encapsulated in Calcium-Alginate Hydrogelated Matrix: Viability and Stability over Aerobic Storage and Simulated Gastrointestinal Conditions

**DOI:** 10.3390/gels9110869

**Published:** 2023-11-01

**Authors:** Daniela Machado, Mariana Fonseca, Rita Vedor, Sérgio Sousa, Joana Cristina Barbosa, Ana Maria Gomes

**Affiliations:** Universidade Católica Portuguesa, CBQF—Centro de Biotecnologia e Química Fina—Laboratório Associado, Escola Superior de Biotecnologia, Rua Diogo Botelho 1327, 4169-005 Porto, Portugal; dmachado@ucp.pt (D.M.); s-marfonseca@ucp.pt (M.F.); rvedor@ucp.pt (R.V.); sdsousa@ucp.pt (S.S.); jcbarbosa@ucp.pt (J.C.B.)

**Keywords:** *Akkermansia muciniphila*, calcium-alginate hydrogelated matrix, encapsulation, extrusion, gastrointestinal passage, probiotic, storage, viability

## Abstract

*Akkermansia muciniphila* is considered a next-generation probiotic to be incorporated in new food and pharmaceutical formulations. Effective delivery systems are required to ensure high probiotic viability and stability during product manufacture, shelf-life, and post-consumption, namely, throughout digestion. Hydrogelated matrices have demonstrated promising potential in this dominion. Hence, this work aimed to evaluate the effect of a calcium-alginate hydrogelated matrix on *A. muciniphila* viability during 28-days refrigerated aerobic storage and when exposed to simulated gastrointestinal conditions, in comparison with that of free cells. *Akkermansia muciniphila* was successfully encapsulated in the calcium-alginate matrix via extrusion (60% encapsulation yield). Furthermore, encapsulated *A. muciniphila* exhibited high stability (a loss in viability lower than 0.2 log-cycle) after 28-days of refrigerated aerobic storage, maintaining its viability around 10^8^ CFU/g. Prominently, as the storage time increased, encapsulated *A. muciniphila* revealed higher viability and stability regarding in vitro gastrointestinal conditions than free cells. This suggests that this encapsulation method may attenuate the detrimental effects of prolonged aerobic storage with a subsequent gastrointestinal passage. In conclusion, encapsulation via extrusion using a calcium-alginate hydrogelated matrix seems to be a promising and adequate strategy for safeguarding *A. muciniphila* from adverse conditions encountered during refrigerated aerobic storage and when exposed to the gastrointestinal passage.

## 1. Introduction

Probiotics are widely known as live microorganisms whose adequate consumption confers health-promoting effects on the host [1]. Traditionally, the microorganisms belonging to *Bifidobacterium* and former *Lactobacillus* genera (recently reclassified into 25 genera [2]) are recognized as well-studied and the most commercialized probiotics [3]. However, the growing trend and expansion of the probiotic market have led to a continuous search for the diversification of the available products. Taking this into account, several studies aiming at the selection of novel strains with different and specific functional properties have been conducted [4]. In this alignment, various bacterial species isolated from human gut microbiota, such as *Akkermansia muciniphila* and *Faecalibacterium* species, have been considered as novel probiotic candidates, also known as next-generation probiotics [3].

In the microbiology field, *A. muciniphila* is typically described as an oval-shaped, anaerobic, Gram-negative, and mucin-degrading bacterium that accounts for approximately 1 to 3% of the total fecal microbiota of healthy adults [5,6]. Recent evidence demonstrated that *A. muciniphila* plays a key role in the preservation of gut barrier integrity, modulation of the host immune response, and improvement of several metabolic pathways, making it a promising therapeutic tool in several metabolic, cardiovascular, neurological, and oncological disorders [7]. For *A. muciniphila* to be applied as a probiotic, effective delivery systems must be developed to guarantee the survival of this novel probiotic during manufacturing, the distribution chain, and shelf-life/storage. Additionally, these strategies must ensure protection throughout the gastrointestinal passage to guarantee that probiotic bacteria reach the intestine (target site) in adequate amounts to exert the desired health benefits [3].

Encapsulation techniques have been proposed as effective strategies to protect probiotic microorganisms against adverse conditions, as these methods may facilitate the controlled release and successful delivery of probiotics to the site of action [8]. Typically, it is defined as an entrapment process of substances (active ingredients) within another material (encapsulant). Several polymers may be used for probiotic encapsulation, including gelatin, chitosan, and alginate, the latter being one of the most commonly applied biomaterials. Alginate is a polyanionic polysaccharide, composed of (1–4)-linked β-D-mannuronate and C-5 epimer α-L-guluronate (G), which exhibits interesting properties in terms of a non-toxic nature, biocompatibility, high hydrophilicity, biodegradability, easy handling, being low in cost, a capability to form a strong gel structure through ionic crosslinking with calcium ions, and pH responsiveness (i.e., it is stable at lower pH levels and unstable at higher pH conditions which is advantageous in tailoring release profiles) [8,9]. Among the various encapsulation techniques, extrusion is recognized as the oldest and most popular methodology to encapsulate probiotics given its attractive characteristics, namely, simplicity, straightforwardness, employment of gentle conditions (without the involvement of organic/harmful solvents and extreme temperatures or pH values), low operational costs, and high probiotic viability [8]. In the extrusion process, probiotics are added and mixed into a biopolymer solution. Subsequently, the suspension is placed in an extruder (pilot scale) or a syringe needle (laboratory scale), which drips off into a hardening solution (most frequently calcium chloride) under gentle stirring [8]. Indeed, previous studies demonstrated that the entrapment of probiotic bacteria in calcium-alginate capsules offered protection against harsh conditions encountered during storage or when subjected to in vitro gastrointestinal conditions [10,11]. Based on this rationale, the present study aimed to evaluate the viability and stability of *A. muciniphila* DSM 22959 entrapped in calcium-alginate capsules produced via extrusion during refrigerated aerobic storage and when exposed to an in vitro gastrointestinal passage.

## 2. Results and Discussion

### 2.1. Capsules Morphology and Encapsulation Yield of Calcium-Alginate Capsules Entrapping Akkermansia muciniphila

Calcium-alginate hydrogelated capsules were selected as a strategy to study the viability and stability enhancement of *A. muciniphila* because, in general, these have good thermal stability and mechanical strength to withstand eventual food processing (mixing) and physiological digestion (passage through gastric conditions to finally release their content in the intestine).

**Figure 1:**
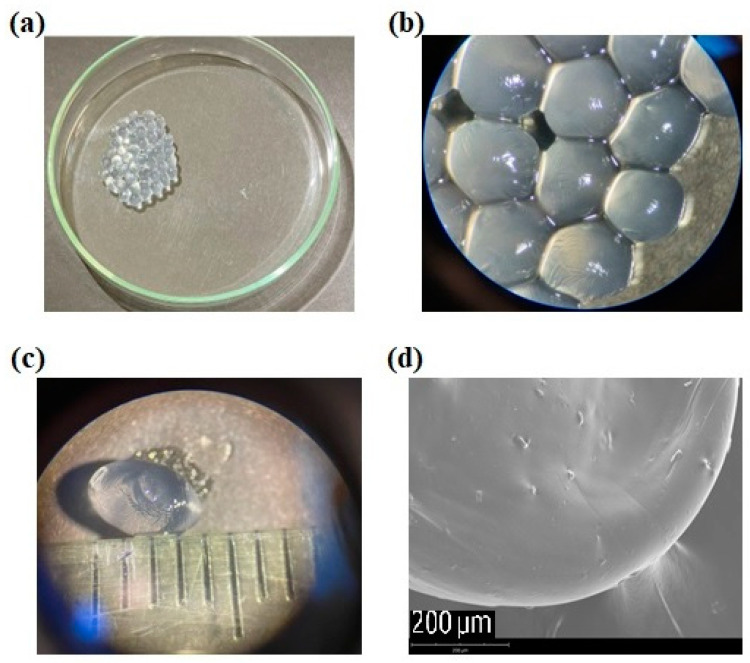
*Akkermansia muciniphila*-loaded capsules observed (**a**) under naked eye; (**b**) grouped under optical microscopy (magnification 20×); (**c**) individualized under optical microscopy (magnification 20×); (**d**) under scanning electron microscopy (magnification: 700×).

Figure 1 shows the morphology of the *A. muciniphila*-loaded calcium-alginate hydrogelated capsules analyzed macroscopically under the naked eye (Figure 1a), under optical microscopy (Figure 1b,c), and scanning electron microscopy (Figure 1d). Morphologically, *A. muciniphila* capsules were presented mostly as spherical structures, with a continuous surface (Figure 1d), estimating a size of approximately 4 mm (Figure 1c).

The encapsulation yield (EY) was 60% (±18%), calculated considering the initial *A. muciniphila* colony-forming units (CFU) number (suspension used for extrusion; 2.10 × 10^10^ CFU) and the final CFU number (calcium-alginate capsules; 1.26 × 10^10^ CFU). This value indicates that this encapsulation technique is suitable for *A. muciniphila* entrapment since the order of magnitude of the cell density (10^10^ CFU) was maintained. After comparing the obtained EY with other studies using similar encapsulation techniques but different probiotic strains, it falls within the reported range. For example, a study conducted by Amine et al. achieved EY values ranging from 31% to 65% for encapsulating *Bifidobacterium longum* ATCC 15708 using an extrusion procedure with sodium alginate and/or O-palmitoylated alginate at various concentrations [12]. Additionally, Frakolaki and coworkers implemented encapsulation via extrusion for *Bifidobacterium animalis* subsp. *lactis* BB-12^®^ using alginate alone or in combination with other encapsulating agents, obtaining EY values ranging from 58.6% to 100%, depending on the employed encapsulating matrix [13].

Notably, van der Ark et al. were the pioneers in *A. muciniphila* encapsulation when these researchers reported an EY of 97.5% using a water-in-oil-in-water double emulsion technique. However, the encapsulated *A. muciniphila* suffered sharp viability reduction after 3 days of storage at 4 °C either under aerobic or anaerobic conditions [14]. Afterward, Marcial-Coba and colleagues encapsulated *A. muciniphila* DSM 22959 in a xanthan and gellan gum matrix, via the extrusion method, with a subsequent freeze-drying step, reporting an encapsulation efficiency ranging from 12.1% to 76.2%, depending on the employed cryoprotective agents [15]. Recently, Almeida and collaborators immobilized *A. muciniphila* DSM 22959, with a 64.4% entrapment efficacy, in a dual hydrocolloid matrix of alginate and denatured whey protein isolate by the emulsification/internal gelation method [16]. While the wide variety of encapsulating materials and techniques may explain the distinct values obtained in this work and in those described in the literature, our results suggest that the extrusion technique in the calcium-alginate matrix seems to be an efficient technological strategy for the entrapment of *A. muciniphila.*

### 2.2. Viability of Akkermansia muciniphila in Encapsulated and Free Forms during Refrigerated Aerobic Storage

**Figure 2:**
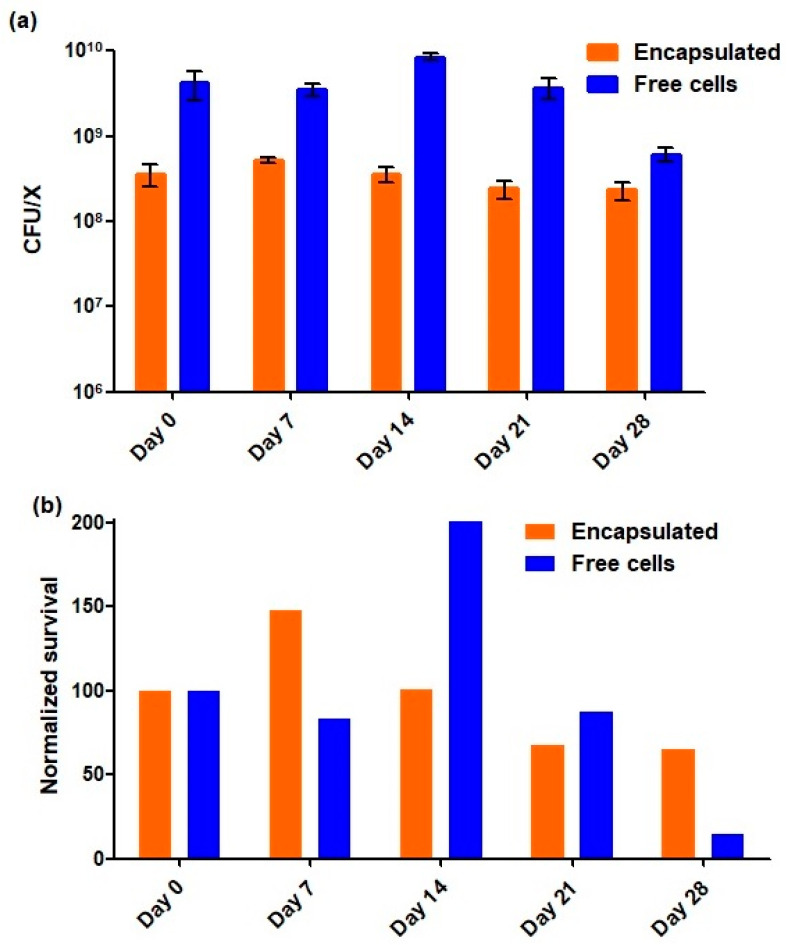
Viability of encapsulated (orange bars) and free cells (blue bars) of *A. muciniphila* DSM 22959 throughout 28 days of refrigerated aerobic storage (**a**) expressed in CFU/X in which X correspond to grams (g) and milliliters (mL) for encapsulated and free cells, respectively, and (**b**) expressed in terms of normalized survival in which the values of starting point (Day 0) were normalized to 100, and the subsequent time points were represented as ratios relative to this starting point.

Although there is no consensus regarding the minimum effective probiotic dose, it is usually accepted that probiotic products should have a minimum concentration of 10^6^ CFU/mL or per gram and that a total of 10^8^–10^9^ probiotic microorganisms should be consumed daily to elicit health benefits [17]. For this reason, one of the major aspects when developing probiotic formulations refers to the ability of these to ensure the maintenance of probiotic viability throughout the manufacturing procedure and chain distribution until it reaches the consumer [3]. Additionally, in the literature, a refrigeration temperature of 4 °C has been related with a high viability level of *A. muciniphila*, either in free or encapsulated forms [15,16,18]. Taking this into account, free and encapsulated *A. muciniphila* were stored at 4 °C under aerobic conditions and their viability was assayed, at specific timepoints, for 28 days. As it can be observed in Figure 2a,b, encapsulated *A. muciniphila* exhibited a high stability in viability (loss lower than 0.2 log-cycle that corresponds to a normalized survival percentage of around 65%, see Figure 2b) after 28 days of refrigerated aerobic storage, maintaining its viability within the magnitude of 10^8^ CFU/g. In contrast, free cell numbers decreased by approximately 1 log-cycle (corresponding to a normalized survival percentage of around 15%, see Figure 2b) within the same period. Notably, the present results concerning encapsulated *A. muciniphila* viability and stability throughout refrigerated aerobic storage contrast positively with those reported in the literature up to the present moment. In fact, van der Ark et al. reported a sharp viability reduction in *A. muciniphila* encapsulated in a water-in-oil-in-water double emulsion when stored at 4 °C for 72 h in both atmospheric conditions: anaerobiosis and aerobiosis [14]. Later, Marcial-Coba and colleagues evaluated the viability of *A. muciniphila* encapsulated via extrusion in a xanthan and gellan gum matrix with subsequent freeze-drying, throughout 30 days, in both aerobic and anaerobic storage conditions at temperatures of 4 °C and 25 °C. These researchers reported a significant decrease (of at least 0.5 log-cycle) in the viability of the freeze-dried microencapsulated *A. muciniphila* after 30 days of storage under both anaerobic and aerobic conditions, at both 4 °C and 25 °C, when compared with the initial concentration [15]. Chang and colleagues encapsulated *A. muciniphila* in a matrix of succinate-grafted alginate doped with epigallocatechin-3-gallate via spray-drying. The authors observed a protective effect of the matrix on the bacterial viability when comparing with the free cells; however, these observations are only verified for 12 days of anaerobic and refrigerated (4 °C) storage [19]. Another investigation conducted by Barbosa and coworkers explored the spray-drying encapsulation technique with different dairy-based matrices to enhance the viability of *A. muciniphila* over aerobic storage. The results indicated that the viability remained around 10^7^ CFU/g up to 28 days at 4 °C under aerobic conditions, using a 10% skim milk matrix [18]. More recently, Almeida and colleagues encapsulated *A. muciniphila* in a dual hydrocolloid matrix containing alginate and denatured whey protein isolate by emulsification/internal gelation and they assessed the viability of the encapsulated bacteria and free counterpart over 95 days of refrigerated storage under aerobic and anaerobic conditions. These researchers reported that during the initial 30 days of refrigerated storage in both atmospheres, there was a similar reduction in the viability of both free and encapsulated cells. However, after 95 days of storage, the viability of the encapsulated *A. muciniphila* experienced a sharper decrease in both anaerobic and aerobic conditions [16]. Thus, our results suggest that extrusion in the calcium-alginate matrix ensures the maintenance of the viability of encapsulated *A. muciniphila* cells at levels around 10^8^ CFU/g during 28 days under feasible household storage conditions, namely aerobic storage at 4 °C. However, the size of the capsules produced via extrusion is relatively high. For instance, the capsules’ size produced herein was estimated at approximately 4 mm (Figure 1c). In the literature, the high dimension/size of the capsules obtained via extrusion is pointed to as one of the main drawbacks of this encapsulation technique [8]; nevertheless, such a drawback may be overcome by adapting their nature to the type of delivery food matrix.

### 2.3. Survival of Akkermansia muciniphila in Encapsulated and Free Forms When Exposed to In Vitro Simulated Gastrointestinal Conditions

It has been postulated that to elicit its beneficial effect, a probiotic microorganism must reach the target site in adequate viable numbers [1]. Therefore, it is important to ensure that delivery systems enclosing probiotic strains are resistant to the adverse gastrointestinal conditions, thus allowing the delivery of the probiotic strain in the optimal required conditions to the target site to trigger the expected benefits. In the present study, the survival of encapsulated and free *A. muciniphila* cells was assessed under simulated gastrointestinal conditions in the following timepoints: 1 and 28 days of refrigerated aerobic storage.

As presented in Table 1, on day 1 of storage, both encapsulated and free *A. muciniphila* maintained viability throughout the in vitro gastrointestinal passage, observing the maintenance of the order of magnitude with a viability of 10^8^ CFU/g and 10^9^ CFU/mL, respectively. In fact, in the literature it has been reported that *A. muciniphila* in its free form exhibits a certain resilience when exposed to gastrointestinal conditions [16,20]. This natural resilience observed in the early stage (at day 1) may be related with the reduced activity of bile salts against Gram-negative bacteria [21] and the presence of an acid resistance system on *A. muciniphila* cells [22].

Regarding the 28 days of storage, when exposed to the in vitro digestion protocol, the encapsulated bacteria reduced the number of viable cells by around 1 log-cycle (achieving a viability level in 10^7^ CFU/g magnitude), while the free cells recorded a viability reduction of higher than 2 log-cycles (viability lower than 8 × 10^5^ CFU/mL). These results showed a higher stability of encapsulated bacteria throughout the gastrointestinal passage compared to its free counterpart at the timepoint of 28 days of storage. In this alignment, a study conducted by Almeida et al. demonstrated that as the storage time increased, *A. muciniphila* encapsulated in an alginate of a denatured whey protein isolate matrix via emulsification/internal gelation showed higher stability when exposed to the gastrointestinal passage than its free counterpart. Specifically, these researchers recorded viability reductions in the free and encapsulated *A. muciniphila* of ca. 2 and lower than 1 log-cycle, respectively, at the timepoint of 30 days of refrigerated aerobic storage [16]. Still, in this context, Barbosa and coworkers showed that the encapsulation of *A. muciniphila* via spray-drying in 10% skim milk using inlet/outlet temperatures of 150/65 °C mitigated the detrimental effects of extended refrigerated aerobic storage for up 60 days, with the subsequent gastrointestinal passage allowing probiotic survival at levels of at least 10 ^7^ CFU/g [18]. Thus, our results suggest that the calcium-alginate capsules produced via extrusion seem to be a promising and adequate strategy to deliver *A. muciniphila* in an intestinal environment at recommended viability levels for probiotic products (above 10^6^ CFU/g). Although the parameters related to *A. muciniphila* release from the capsules were not assayed, a possible mechanism of releasing these probiotic cells may be hypothesized. In the literature, it is described that alginate confers gastric-acid-protective effects on encapsulated probiotics [23,24]. In fact, the alginate capsules tend to shrink at a low pH (such as in a gastric environment), which prevents the release of encapsulated probiotic cells [8,25]. Once it passes to the higher pH of the intestinal environment, the alginate becomes a soluble alginic acid layer, allowing the release of probiotic cells [8].

## 3. Conclusions

This study showed that this extrusion technique, using a calcium-alginate encapsulating matrix, is an interesting and adequate strategy for *A. muciniphila* delivery. Indeed, this encapsulation technique guaranteed the efficient production of high-loaded capsules that maintained probiotic viability in a magnitude of 10^8^ CFU/g throughout refrigerated aerobic storage for 28 days. Additionally, as the storage time increased, encapsulated *A. muciniphila* revealed higher viability and stability regarding in vitro gastrointestinal conditions than free cells. This finding suggests that this encapsulation procedure may attenuate the detrimental effects of prolonged aerobic storage with a subsequent gastrointestinal passage. At the same time, this extrusion method ensured *A. muciniphila* delivery at levels required for probiotic products (above 10^6^ CFU/g), even after 28 days of refrigerated aerobic storage with a subsequent in vitro gastrointestinal passage. To enable the consolidation of the potential application of these probiotic calcium-alginate capsules in suitable food and pharmaceutical products, further studies on their size and morphology are required. Also, additional work assessing the viability and stability of encapsulated *A. muciniphila* under longer aerobic storage periods should be performed, to simulate the storage conditions of commercial probiotic products more realistically. Likewise, the rate and time of *A. muciniphila’s* release from capsules should be determined to evaluate if a controlled release of *A. muciniphila* occurs and, consequently, its efficient delivery to the target site (intestine).

## 4. Materials and Methods

### 4.1. Bacterial Strain and Culture Conditions

Freeze-dried *A. muciniphila* DSM 22959 strain obtained from DSMZ collection (Leibniz Institute DSMZ—German Collection of Microorganisms and Cell Cultures, Braunschweig, Germany) was used in present study. For long-term storage, this strain was maintained frozen at −80 °C in PYG broth supplemented with 0.1% (*m*/*v*) mucin [PYGM; media composition as recommended by DSMZ [26] except that no resazurin was added], with 20% (*v*/*v*) glycerol (Fisher Scientific, Loughborough, UK). For each experiment, a glycerol stock of *A. muciniphila* DSM 22959 was thawed and grown in PYGM broth at 37 °C for 20–24 h under anaerobic conditions (85% N_2_, 5% H_2_, and 10% CO_2_) achieved in an anaerobic incubator (Whitley A35 HEPA anaerobic workstation, Bingley, UK). The bacterial cultures were then propagated, at least two subsequent culturing steps, by inoculating fresh medium (PYGM broth) at 10% (*v*/*v*) and incubating under same growth conditions. The resulting cultures were centrifuged at 12,000× *g* for 30 min at 4 °C (Sorvall LYNX 4000 Superspeed Centrifuge, Thermo Fisher Scientific, Waltham, MA, USA) and washed once with the same volume of physiological saline solution (NaCl at 0.85% *m*/*v*). After centrifugation in the same previous conditions, the pelleted biomass was resuspended in physiological saline solution to reach a final cell concentration around 10^9^ CFU/mL. This resulting bacterial suspension was either used directly as free cells control or for encapsulation procedure via extrusion (5 mL).

### 4.2. Extrusion Procedure

Encapsulation via the extrusion technique was based on Sousa and colleagues’ protocol where optimum conditions were already adapted for different commercial probiotic strains [11]; herein, the protocol underwent some modifications. Briefly, *A. muciniphila* saline suspension (with a concentration of around 10^9^ CFU/mL) was added at 10% (*v*/*v*) to 2% (*m*/*v*) sodium alginate with molecular weight ranging from 12,000–40,000 Daltons (Sigma-Aldrich, St. Louis, MO, USA). Afterwards, the alginate–culture mixtures (in a proportion of 45 mL alginate:5 mL bacterial suspension) were loaded in a 60 mL syringe (Enfa, Jiangsu Kanghua Medical Equipment Co., Ltd., Changzhou, China) coupled with a syringe needle (21 G × 1.5″, ICOplus 3, KD Medical GmbH Hospital Products, Berlin, Germany). This mixture (50 mL) was then extruded into 200 mL of 4% (*m*/*v*) CaCl_2_ solution and stirred at 200 rpm (Heidolph MR 3001 magnetic stirrer; Heidolph Instruments GmbH and Co. KG, Schwabach, Germany). The extrusion rate was 4.0 mL/min. The flow rate was controlled using a syringe pump (Braintree Scientific BS-300 syringe pump, Braintree Scientific, Braintree, MA, USA). Afterward, the resulting capsules were left in contact with the CaCl_2_ solution for 30 min at room temperature to ensure complete solidification. The CaCl_2_ solution was subsequently removed by decanting and the capsules were suspended in physiological saline solution. Lastly, the capsules were recovered using a fine mesh strainer and they were stored according to conditions described in Section 4.6.

### 4.3. Enumeration of Free and Encapsulated Akkermansia muciniphila Cells

For the enumeration of free *A. muciniphila* cells, decimal dilutions were performed in PBS and spotted, in triplicate, on PYGM agar plates [PYGM broth supplemented with 1.5% (*m*/*v*) agar (BIOKAR Diagnostics, Beauvais, France)]. Plates were incubated for 5–7 days at 37 °C under anaerobic conditions, and results were expressed in CFU/mL. Concerning the encapsulated bacteria, the capsules were suspended in a tri-sodium citrate dihydrate (Merck KGaA, Darmstadt, Germany) solution at 2.28% (*m*/*v*) in a 1:9 (g/mL) ratio and subjected to the mechanical action of sterile pellet pestle (Sigma-Aldrich, St. Louis, MO, USA) assisted with vortexing for 5 min to allow the complete release of *A. muciniphila* cells from capsules. The resulting suspension was then serially diluted, as described for the free cells, and the results were expressed as CFU/g.

### 4.4. Encapsulation Yield Calculation

Encapsulation yield (EY) is a combined measurement of the efficacy of entrapment and survival of viable cells during the encapsulation procedure, and it was calculated according to the formula proposed by Martin and coworkers [27]:

EY (%) = (N/N0) × 100,(1)

where N is the number of CFU released from capsules and N0 is the number of CFU present in bacterial suspension added to the alginate solution during the encapsulation procedure.

### 4.5. Capsules Morphology

The appearance of *A. muciniphila*-loaded capsules was analyzed under optical microscopy using an Olympus Stereo Microscope SZ60 (Olympus, Tokyo, Japan). Furthermore, capsules morphology was assessed through scanning electron microscopy. Initially, alginate capsules were fixed with a 2.5% (*v*/*v*) glutaraldehyde solution for 1 h. The samples were then washed with water, and dehydrated through immersion in a graded ethanol series (30, 50, 70, 80, 90, and 100% (*v*/*v*) for a minimum of 10 min each). Afterwards, capsules were swiftly dried by dripping hexamethyldisilazane on top and immediately evaporating under a gentle stream of nitrogen. The capsules were placed on observation pins, covered with double-sided adhesive carbon tape (NEM tape; Nisshin, Tokyo, Japan), sputter-coated with gold/palladium, and visualized using a Phenom XL G2 (Thermo Fischer Scientific, Waltham, MA, USA) scanning electron microscope. The micrographs were obtained at an accelerating voltage of 15 kV, utilizing the secondary electron detector.

### 4.6. Viability of Free and Encapsulated Akkermansia muciniphila Cells during Refrigerated Aerobic Storage

The effect of encapsulation via extrusion on *A. muciniphila* viability was assayed, in comparison to free cells, throughout refrigerated aerobic storage for 28 days. Free cells were stored as follows: equal portions (1 mL) of cell suspension were stored at 4 °C under aerobic conditions into 10 sterile microtubes, corresponding to two replicas for each sampling timepoint. Concerning the encapsulated bacteria, capsules were weighed into 50 mL sterile centrifuge tubes and suspended in physiological saline solution in a 1:9 (g/mL) ratio; these preparations were performed in duplicates for each sampling timepoint, and subsequently stored at 4 °C under aerobic conditions. The viability of free and encapsulated *A. muciniphila* cells was evaluated on the day of encapsulation and after 7, 14, 21, and 28 days of refrigerated aerobic storage, following the protocol previously described in Section 4.3.

### 4.7. Survival of Free and Encapsulated Akkermansia muciniphila Cells in In Vitro Simulated Gastrointestinal Passage

The viability of free and encapsulated *A. muciniphila* cells when exposed to in vitro simulated gastrointestinal conditions was determined 1 and 28 days after the extrusion procedure using a standardized digestion method [28] with minor changes. Briefly, either 0.5 mL of free cells in 0.85% (*m*/*v*) NaCl or 0.5 g of calcium-alginate capsules were distributed into independent tubes (two replicates per timepoint and condition). To simulate the temperature and peristaltic movements of the human digestion, an orbital shaker incubator (Wiggen Hauser, Berlin, Germany) was used at 37 °C and 200 rpm. For each assay, all enzyme solutions were freshly prepared. For the gastric phase, samples were exposed to 2 mL of simulated gastric fluid (pH 3) containing pepsin (2000 U/mL—from porcine gastric mucosa; Sigma Aldrich, St. Louis, MO, USA) for 2 h. Afterward, intestinal conditions were simulated for 3 h at pH 7 by adding 4 mL of simulated intestinal fluid containing pancreatin (based on the trypsin activity at 100 U/mL in the final mixture; Sigma Aldrich, St. Louis, MO, USA) and bile salts (Sigma Aldrich, St. Louis, MO, USA). To evaluate the effect of gastric and intestinal conditions on *A. muciniphila* viability (free and encapsulated forms), samples were collected at the end of each phase (gastric and intestinal) and cell enumeration was performed according to the procedure previously described in Section 4.3. Note that in vitro digestion protocol was performed under an aerobic atmosphere, while the PYGM agar plates were incubated under anaerobic conditions.

## Figures and Tables

**Table 1:** Evolution of viable cell numbers of *A. muciniphila* DSM 22959 in encapsulated (CFU/g) and free (CFU/mL) forms during in vitro gastrointestinal passage at 1 and 28 days of refrigerated aerobic storage. Data are shown as the mean ± standard deviation.

Condition	Day 1		Day 28	
	Encapsulated	Free	Encapsulated	Free
Initial Concentration	(3.82 ± 1.26) × 10^8^	(3.40 ± 0.39) × 10^9^	(2.32 ± 0.55) × 10^8^	(6.12 ± 1.11) × 10^8^
After gastric phase	(2.74 ± 1.17) × 10^8^	(2.57 ± 0.31) × 10^10^	(1.15 ± 0.12) × 10^8^	(7.46 ± 6.91) × 10^6^
After intestinal phase	(1.57 ± 0.33) × 10^8^	(4.83 ± 0.36) × 10^9^	(2.93 ± 1.09) × 10^7^	<LOD ^1^

^1^ LOD means limit of detection, corresponding in CFU plating technique to 8 × 10^5^ CFU/mL or /g.

## Data Availability

The data presented in this study are available in the article.
